# UNTIL IT BURSTS OR ALL OF US BURST. A SCHIZOTYPICAL CASE.

**DOI:** 10.1192/j.eurpsy.2023.2055

**Published:** 2023-07-19

**Authors:** B. Rodríguez Rodríguez, N. Navarro Barriga, M. Fernández Lozano, M. J. Mateos Sexmero, M. A. Andreo Vidal, M. Calvo Valcárcel, P. Martínez Gimeno, M. P. Pando Fernández, A. Aparicio Parras, M. D. L. Á. Guillén Soto, T. Jiménez Aparicio, M. D. C. Vallecillo Adame, C. de Andrés Lobo, A. A. Gonzaga Ramírez, G. Guerra Valera, M. Queipo de Llano de la Viuda, M. Esperesate Pajares

**Affiliations:** 1 Psichiatry, HCUV, Valladolid, Spain

## Abstract

**Introduction:**

Schizotypal disorder is conceptualized as a stable personality pathology (Cluster A) and as a latent manifestation of schizophrenia. It can be understood as an attenuated form of psychosis or high-risk mental state, which may precede the onset of schizophrenia or represent a more stable form of psychopathology that doesn’t necessarily progress to psychosis.

**Objectives:**

To exemplify the continuum of psychosis

**Methods:**

Review of scientific literature based on a relevant clinical case.

**Results:**

39-year-old male living with his parents. He started studying philosophy. He is a regular cannabis user and has an aunt with schizophrenia. He’s admitted to psychiatry for behavioral disturbance in public. He refers to having been hearing a beeping noise in his street for months, what he interprets as a possible way of being watched due to his past ideology. Without specifying who and why, he sometimes shouts “until it bursts” to stop the noise and he thinks that his neighbours alerted the police about his behavior. During the interview he alludes to Milgram’s experiment, saying that throughout history there have been crimes against humanity and those who pointed them out were labeled “crazy”. His father refers that he has always been “strange” and with certain extravagant revolutionary ideas and thoughts. He doesn’t maintain social relationships and dedicates himself to reading and writing.

**Conclusions:**

It’s important to understand psychosis as a continuum to advance the understanding of etiology, pathophysiology and resilience of psychotic disorders and to develop strategies for prevention and early intervention

**Disclosure of Interest:**

None Declared

